# Genomic prediction of resistance to *Hymenoscyphus fraxineus* in common ash (*Fraxinus excelsior* L.) populations

**DOI:** 10.1111/eva.13694

**Published:** 2024-05-03

**Authors:** Joanna Meger, Bartosz Ulaszewski, Małgorzata Pałucka, Czesław Kozioł, Jarosław Burczyk

**Affiliations:** ^1^ Department of Genetics, Faculty of Biological Sciences Kazimierz Wielki University Bydgoszcz Poland; ^2^ Kostrzyca Forest Gene Bank Miłków Poland

**Keywords:** ash dieback, *Fraxinus excelsior*, genomic prediction, GWAS, *Hymenoscyphus fraxineus*

## Abstract

The increase in introduced insect pests and pathogens due to anthropogenic environmental changes has become a major concern for tree species worldwide. Common ash (*Fraxinus excelsior* L.) is one of such species facing a significant threat from the invasive fungal pathogen *Hymenoscyphus fraxineus*. Some studies have indicated that the susceptibility of ash to the pathogen is genetically determined, providing some hope for accelerated breeding programs that are aimed at increasing the resistance of ash populations. To address this challenge, we used a genomic selection strategy to identify potential genetic markers that are associated with resistance to the pathogen causing ash dieback. Through genome‐wide association studies (GWAS) of 300 common ash individuals from 30 populations across Poland (ddRAD, dataset A), we identified six significant SNP loci with a *p*‐value ≤1 × 10^−4^ associated with health status. To further evaluate the effectiveness of GWAS markers in predicting health status, we considered two genomic prediction scenarios. Firstly, we conducted cross‐validation on dataset A. Secondly, we trained markers on dataset A and tested them on dataset B, which involved whole‐genome sequencing of 20 individuals from two populations. Genomic prediction analysis revealed that the top SNPs identified via GWAS exhibited notably higher prediction accuracies compared to randomly selected SNPs, particularly with a larger number of SNPs. Cross‐validation analyses using dataset A showcased high genomic prediction accuracy, predicting tree health status with over 90% accuracy across the top SNP sets ranging from 500 to 10,000 SNPs from the GWAS datasets. However, no significant results emerged for health status when the model trained on dataset A was tested on dataset B. Our findings illuminate potential genetic markers associated with resistance to ash dieback, offering support for future breeding programs in Poland aimed at combating ash dieback and bolstering conservation efforts for this invaluable tree species.

## 1 INTRODUCTION

Anthropogenic environmental changes have resulted in an increased occurrence of introduced insect pests and pathogens (Fisher et al., 2012). These changes are primarily driven by factors such as climate change (Jung, 2009; Tubby & Webber, 2010), long‐distance transport (Boyd et al., 2013; Guo et al., 2012), and the global trade of living organisms (Santini et al., 2013). The rise in pests and diseases has significant impacts on native species, leading to elevated mortality rates and/or diminished overall health (Dobson, 2009). Among the affected species, long‐lived organisms with slow reproduction rates, such as forest trees, are particularly vulnerable to novel pathogens. Observations of increased mortality in European elm (*Ulmus glabra* Hudson) in Europe (Bartnik et al., 2015), beech (*Fagus sylvatica* L.) in Central Europe (Jung, 2009), chestnut (*Castanea dentata* (Marshall) Borkh.) in North America (Ellison et al., 2005), and various ash species (*Fraxinus* spp.) in Europe and North America (Kowalski, 2006; Orlova‐Bienkowskaja & Volkovitsh, 2015) highlight the need for effective forest management strategies to protect and prevent the further decline of these valuable tree species.

While traditional breeding programs based on phenotypic selection have shown effectiveness in achieving durable resistances against pests and pathogens in trees (Alfaro et al., 2013; Martín et al., 2019, 2021; Sniezko et al., 2020; Sniezko & Koch, 2017), the development of genomic resources for forest trees can enhance tree breeding programs and selection efforts, improving their overall efficiency. Genome‐wide association studies (GWAS) are a robust method for identifying potential causal genes or genomic regions that are associated with plant phenotypic variation (Ingvarsson & Street, 2011; Kruglyak, 2008). Several studies have successfully identified single‐nucleotide polymorphisms (SNPs) that are associated with resistances to abiotic and biotic stresses, including drought and disease resistance, in various plant species (Beaulieu et al., 2020; Bouvet et al., 2020; Cappa et al., 2022; Laverdière et al., 2022; Lenz et al., 2020; Muchero et al., 2018; Mukrimin et al., 2018; Soro et al., 2022; Stocks et al., 2019; Vázquez‐Lobo et al., 2017; Wang et al., 2019; Westbrook et al., 2020). The utilization of GWAS results can expedite the conventional breeding process and enable genotype‐based selection (Neale & Kremer, 2011).

Genomic selection (GS) is a breeding strategy that utilizes a prediction model that has been developed from the genotypic and phenotypic data of a training population, to estimate the genomic estimated breeding values (GEBVs) for individuals in a breeding population based on their genomic profiles, as proposed by Meuwissen et al. (2001). These GEBVs are then used for selecting superior individuals as parents for the next generation in breeding programs. GS offers advantages over traditional breeding methods that rely on phenotypic selection by significantly reducing the duration of the breeding cycle and eliminating the need for extensive field testing of progeny (Heffner et al., 2010). It is especially crucial for traits that manifest late in development (particularly in long‐living species) or that are challenging to evaluate accurately, such as pest and disease resistance. Despite the high genetic diversity and genotyping costs associated with forest trees, GS has shown promising results in studies involving Eucalyptus and loblolly pine (Grattapaglia & Resende, 2011; Resende, Resende et al., 2012; Resende, Muñoz et al., 2012). Recent studies focusing on stress resistance, including diseases, pests, and drought, have also reported encouraging findings regarding the prediction of complex traits in forest trees (Beaulieu et al., 2020; Bouvet et al., 2020; Lenz et al., 2020; Stocks et al., 2019; Westbrook et al., 2020).

Common ash (*Fraxinus excelsior* L.) is a widely distributed deciduous tree species in Europe, extending from the Mediterranean Sea to southern Scandinavia. It plays a significant ecological role in European forests (Hultberg et al., 2020), and it possesses valuable wood properties (Dobrowolska et al., 2011), making it economically important. However, the invasive fungal pathogen *Hymenoscyphus fraxineus* (HF) (T. Kowalski) Baral, Queloz, and Hosoya (Baral et al., 2014), responsible for inducing tree dieback and causing high mortality rates across Europe (Coker et al., 2019), poses a significant threat to the future of ash species. Ash dieback was first identified in the northwest of Poland in 1992 (Kowalski, 2006), and it subsequently spread throughout Europe (McKinney et al., 2014; Pautasso et al., 2013). The characteristic symptoms of this disease include crown damage, with shoot, twig, and branch dieback, early leaf wilting and shedding, the formation of necrotic lesions on leaves and cambium, bark discoloration, and the sprouting of epicormic shoots (McKinney et al., 2014; Skovsgaard et al., 2017). Only a small percentage of common ash genotypes within populations, ranging from 1 to 5 percent, remain undamaged (Kjær et al., 2012; McKinney et al., 2011, 2014; Muñoz et al., 2016; Pliūra et al., 2011; Stener, 2013). Identifying genotypes that show no damage is a research priority to facilitate the selection and conservation of ash populations in European woodlands.

Previous genetic studies of *F. excelsior* have demonstrated heritability values above 0.4 (Enderle et al., 2015; Kjær et al., 2012; Lobo et al., 2014, 2015; McKinney et al., 2011; Muñoz et al., 2016; Pliūra et al., 2011; Stener, 2013) and the polygenic nature of resistance to *H. fraxineus* (Muñoz et al., 2016; Stocks et al., 2019). Several studies have identified genetic markers that are associated with increased tolerance to ash dieback (Chaudhary et al., 2020; Harper et al., 2016; Havrdová et al., 2016; McKinney et al., 2014; Menkis et al., 2020; Sahraei et al., 2020; Sollars et al., 2017; Stocks et al., 2019). These markers can be potentially utilized to identify resistant trees that can serve as the foundation for a breeding program (Plumb et al., 2020).

Studying resistance in adult ash populations is particularly challenging. The response of adult ash trees to HF infection is complex, and the dieback symptoms may appear and worsen for many years. Therefore, the collection of tolerant and susceptible adults for GWAS studies is recommended to be conducted in populations that have been exposed to HF for an extended period, such as those in Poland. The hope is that the ash trees exhibiting a good health status should be confidently considered resilient to HF. However, it cannot be ruled out that resistant individuals were eliminated due to unfavorable environmental conditions. Conversely, favorable environments may have allowed individuals with lower genetic resistance to thrive. In Poland, where selection pressure has been ongoing for more than 30 years, the identification of genetic markers in common ash populations has not been performed yet.

Reduced representation sequencing approaches can effectively decrease genotyping costs while still enabling the detection of SNPs associated with resistance through GWAS analysis, and enhancing the efficiency of genomic selection (Iwata et al., 2016; Kumar et al., 2012; Poland et al., 2012; Varshney et al., 2005). However, the quality of GWAS studies, particularly those utilizing reduced representation sequencing, also relies on the quality of the assembly and annotation of the reference genomes. Most of the existing GWAS studies in ash have relied on the reference genome, BATG 0.5 (Sollars et al., 2017). However, we recently published the new reference genome of *F. excelsior*, accessible at (https://www.ncbi.nlm.nih.gov/data‐hub/genome/GCA_019097785.1/). The genome comprises 23 chromosomes and 415 scaffolds spanning a total of 806.5 Mb. It consists of 58.90% repetitive DNA and includes 41,355 high‐confidence gene models, opening up new opportunities for in‐depth genomic studies.

In this study, we are using a genomic selection strategy employing an optimal panel of SNPs selected through genome‐wide association studies, and we are assessing its effectiveness in predicting ash tree resistance to *H. fraxineus*. This approach was previously successfully conducted by Stocks et al. (2019), where genomic prediction was used to determine the health status of common ash in southeast England. This study identified 3149 SNPs associated with health status, selected from the extremes of ash dieback damage phenotypes. Next, a genomic prediction model was trained using pool‐seq data from 1250 trees, based on 10,000 loci that had the most significant association with disease susceptibility in a pool‐seq GWAS. These 10,000 SNPs were able to predict the disease classification of individually sequenced 150 test trees with 68% accuracy.

In our study, we initially conducted a GWAS using double‐digest restriction site‐associated DNA (ddRAD) data to identify significant SNPs associated with the health status of 300 individuals of common ash from 30 populations across Poland (dataset A). Subsequently, we evaluate the genomic prediction accuracies of the most significant GWAS marker sets in predicting health status, considering two scenarios. Firstly, we perform cross‐validation on dataset A. Secondly, we train markers using dataset A and evaluate their performance on dataset B, which entails whole‐genome sequencing data on 20 individuals from two distinct populations in Poland (dataset B). Additionally, we assess the genomic prediction accuracy for various health status indicators in dataset B, including binary health status, defoliation (%), tree crown vitality, trunk condition, synthetic tree damage index (Syn), and the synthetic variable SynT (the average value of Syn and the trunk condition class after the prior standardization of both variables), using different sets of SNPs. These sets comprise 100, 200, 500, 1000, 2500, 5000, and 10,000 SNPs selected based on GWAS and randomly selected SNPs for both scenarios. Finally, we propose a set of genetic markers and a geographical region that could potentially be supported by breeding programs aimed at enhancing the resistance of ash trees.

## 2 MATERIALS AND METHODS

### 2.1 Plant materials

**FIGURE 1:**
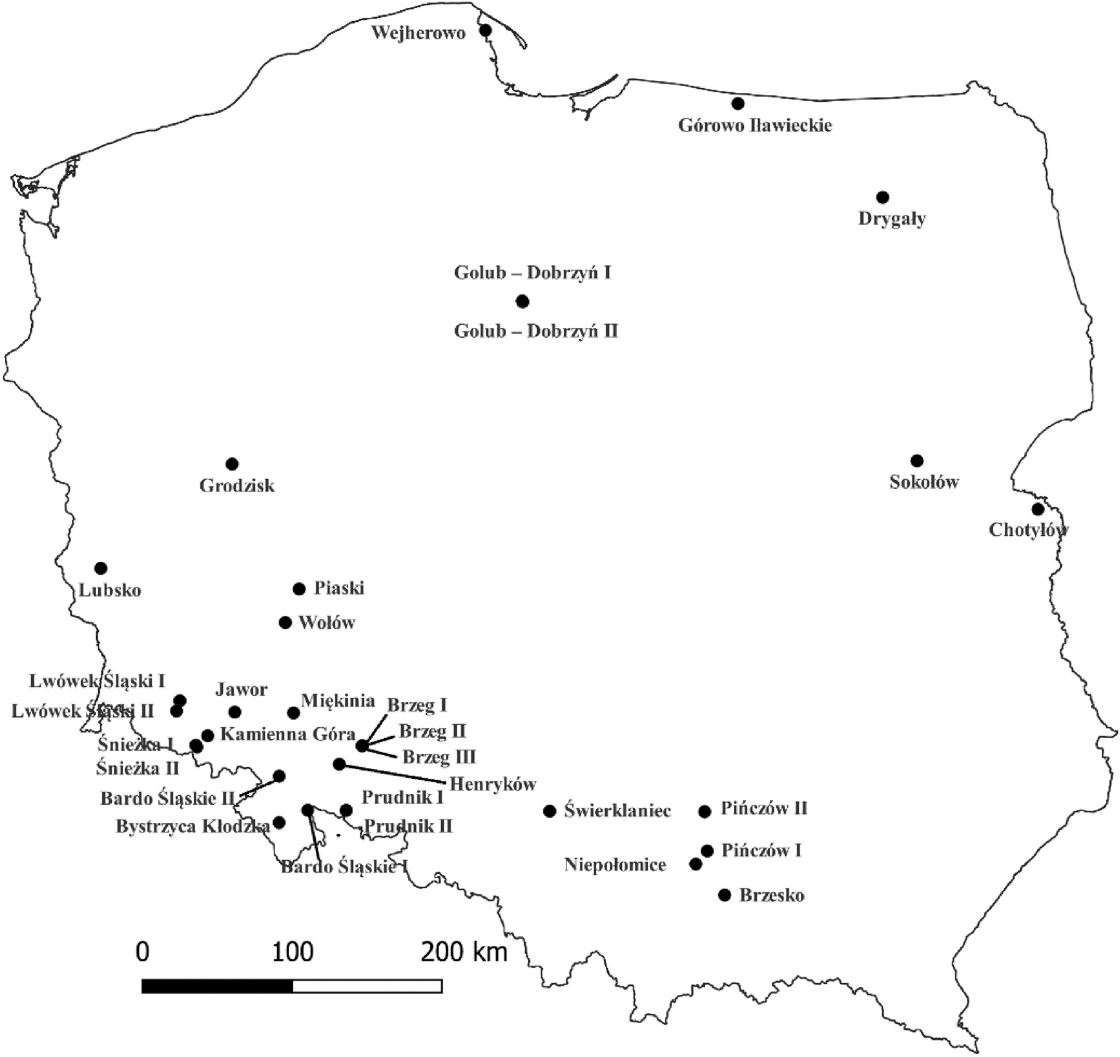
Geographical distribution of the studied populations of common ash in Poland.

This study was based on two datasets. The first one (dataset A) included 300 common ash individuals from 30 populations (10 individuals per population) in Poland (Figure S1, Table S1). The second (dataset B) consisted of 20 individuals from the Drygały and Grodzisk populations (10 individuals per population), each belonging to one of the two evolutionary lineages of *F. excelsior* revealed by chloroplast DNA (Heuertz, Fineschi, et al., 2004; Heuertz, Hausman, et al., 2004; Meger et al., 2024) (Figure 1, Table S1).

We selected populations with a high natural infection pressure by *H. fraxineus*, where apart from the affected trees, there were also trees that did not exhibit any visible disease symptoms. Within each population, consisting of 40 to 50 individuals, five healthy and five diseased trees were selected. An exception was observed in the Drygały and Grodzisk populations, where we found only four and two healthy trees, respectively, while the remaining ones were diseased. All of the analyzed trees were over 70 years old. Fresh leaves were collected in June/July 2017 for dataset A, and in July 2022 for dataset B.

### 2.2 Phenotypic data

The phenotypic data for both datasets are provided in Table S1. Each sampled individual from both datasets was rated for their ash dieback damage according to two groups: 0 for a healthy tree with no visible damage by *H. fraxineus*, and 1 for a strongly damaged tree.

For 20 trees from dataset B, defoliation, tree crown vitality, and trunk condition were additionally assessed. The defoliation of trees was estimated as the percentage of leaf canopy loss from visual expectation from the ground. Defoliation was assessed in steps of 5% increments, starting from 0%, up to 95%. Trees with 100% defoliation were not considered.

The crown vitality was assessed according to Roloff's classification (Roloff, 1988, 2001), based on the lengths and types of shoots growing in the top of the crown. Trees were classified into four groups of damage:

- Class 0 – a live, undamaged tree – the crown shape is spherical without gaps, with long shoots dominating; symmetrical shoots occur, and multi‐year branches have a “fan” shape (long shoots come from the axis of the main top shoots and the side branches).
- Class 1 – weakened tree – the upper part of the crown has gaps between the branches of the “lance” type (the main axes of the shoots form shortened long shoots, and the side branches are short shoots), while the inside of the crown is dense.
- Class 2 – damaged tree – clear thinning of the upper and middle parts of the crown, “claw” type branches (the main axes of the top shoots only produce short shoots, forming bending “chains of short shoots” over the years), and single thick branches.
- Class 3 – strongly damaged, dying tree – the crown shape is irregular, with the presence of thick and dying branches, and “claws.”

The synthetic tree damage index (Dmyterko & Bruchwald, 1998) for each tree was calculated from the combination of defoliation and vitality, according to the following formula:

$$\mathrm{Syn}=\frac{0.03*\mathrm{Def}+\mathrm{Vit}}{2},$$

where Syn, synthetic tree damage index; Def, tree defoliation (%); Vit, vitality.

The synthetic tree damage index is a continuous variable that takes in values within the range of 0 to 3. Based on this, four degrees of tree damage are distinguished:

- Class 0 – Syn ≤0.5 – undamaged tree,
- Class 1 – 0.5 < Syn ≤1.5 – slightly damaged tree,
- Class 2 – 1.5 < Syn ≤2.5 – moderately damaged tree,
- Class 3 – Syn >2.5 – strongly damaged tree.

The condition of the trunk was assessed, based on the following classification of three groups: 0 – for a healthy trunk with no visible damage, 1 – for single necrotic lesions at the base of the trunk, 2 – for visible trunk rot.

The last index of assessing tree damage (total synthetic tree damage – SynT) was created from the average value of the synthetic tree damage class (Syn) and the trunk condition class after the prior standardization of both variables. An increase in the SynT value indicates an increase in the overall level of damage to trees.

### 2.3 DNA extraction and sequencing

Total DNA was extracted from the young leaves of 300 sampled individuals from dataset A using the GeneMATRIX Plant & Fungi DNA Purification Kit (EURx, Poland), following the manufacturer's instructions. Extracted DNA was quantified using the Eppendorf BioPhotometer and Quantus Fluorometer (Promega). Genomic DNA was digested with *SphI* and *MboI*. Library preparation and double‐digest restriction site‐associated DNA (ddRAD) sequencing using an Illumina HiSeq 2500 platform with 125 bp paired‐end reads were performed at IGA Technology Services (Udine, Italy).

DNA extraction from 20 individuals from dataset B was based on the method described by Wang et al. (2012). The quality of the isolated genomic DNA was checked using both a Quantus Fluorometer (Promega) and gel electrophoresis. Library preparation using Truseq PCR‐free with 350 bp inserts, and whole‐genome sequencing using NovaSeq6000 with 150 bp paired‐end reads was performed by Macrogen (Amsterdam, The Netherlands).

### 2.4 SNP genotyping data

Sequence data were assessed with FastQC software (Andrews, 2010). Raw read sequences from ddRAD (dataset A) were processed for adapter removal using Cutadapt software (Martin, 2011); contaminants (e.g., chloroplast genome sequences) were filtered with the package ERNE‐FILTER (Del Fabbro et al., 2013). The raw sequence reads obtained from whole‐genome sequencing (dataset B) underwent a trimming process to eliminate low‐quality segments and adapters. This trimming procedure utilized Trimmomatic‐0.33 (Bolger et al., 2014) and involved quality trimming using a sliding window with a window size of four base pairs and a quality threshold of 15. Any reads with a size less than 36 base pairs were excluded from further analysis.

The reads from both datasets were then aligned to the *F. excelsior* genome assembly FRAX_001_PL (https://www.ncbi.nlm.nih.gov/data‐hub/genome/GCA_019097785.1/) using the Burrows – Wheeler Aligner (BWA – MEM algorithm) with the default settings (Li & Durbin, 2009). SAM files were converted to BAM files, and these were sorted and indexed using SAMtools v.0.1.19 (Li et al., 2009).

GATK 3.5 (DePristo et al., 2011) was used to detect SNPs in each aligned sample for both datasets separately, using a minimum Phred‐scaled confidence threshold of 30. Subsequently, the GATK tools “VariantFiltration” and “SelectVariants” were employed to eliminate low‐quality variants. The specific criteria for exclusion included: QD < 20.0, MQ < 40.0, MQRankSum < −12.5, and ReadPosRankSum < −8.0. The filtering of SNPs was performed using VCFtools (Danecek et al., 2011) to include only biallelic SNPs that were present in at least 95% of individuals, with a minimum depth of 10 reads and a minor allele frequency >5%. SNPs from dataset A were then pruned for linkage disequilibrium (LD), using PLINK 2.0 (Purcell et al., 2007). A cutoff of *r* ^*2*^ > 0.5 was used for removing SNPs showing strong signals of LD.

### 2.5 Genetic structure

The genetic structures of the analyzed populations in datasets A and B (a total of 32 populations) were assessed using two distinct methods. First, we conducted principal component analysis (PCA) with the R package SNPRelate version 1.32.1 (Zheng et al., 2012) and visualized the outcomes with ggplot2 version 3.4.0 (Wickham, 2016). Second, we applied the Bayesian model‐based clustering method with fastSTRUCTURE version 1.0 software (Raj et al., 2014). Our analysis involved testing for *K* values ranging from 1 to 32 clusters, with 10 iterations for each run. The optimal *K* value was determined using the chooseK tool within the package. Visualization of the best results was performed using Clumpak software (Kopelman et al., 2015).

### 2.6 Genome‐wide association study

The genome‐wide association study (GWAS) for the health status of dataset A employed the mixed linear model (MLM) method (Yu et al., 2006). This method was implemented through the “GWAS” function in the R package rrBLUP version 4.6.1 (Endelman, 2011). Both the kinship matrix (*K*) and the first four principal components (PCs) were incorporated into the MLM model to address relatedness among the genotypes and to avoid false positives. The kinship matrix was generated using the “A.mat” function from the R package rrBLUP version 4.6.1 (Endelman, 2011). To correct for multiple testing, the method proposed by Li and Ji (2005) was utilized.

Given that health status is a quantitative trait influenced by numerous genetic loci, the Bonferroni test criterion (0.05/number of SNPs) is overly stringent for use as a threshold. As no SNP reached the Bonferroni threshold, the threshold value of *p*‐value ≤1 × 10^−4^ was arbitrarily set for the following reasons: Firstly, the relatively small sample size may lead to insufficient statistical power. Secondly, the objective of this analysis is to identify the most significant loci associated with health status, which are crucial for subsequent genomic prediction analyses. Manhattan plots visualizing GWAS results were generated using the R package qqman ver. 0.1.8 (Turner, 2014).

### 2.7 Genomic prediction

To evaluate the best genomic prediction accuracy, we employed five regression models, utilizing all SNPs available in dataset A. The dataset was divided into training and testing populations at a 60/40 ratio for assessment. The regression models included genomic best linear unbiased prediction (GBLUP) with ridge kernel regression (RR) or Gaussian kernel regression (GAUSS), Bayesian Ridge Regression (BRR), Bayesian Lasso (BL), and BayesB. GBLUP with RR and GAUSS were implemented using the “kinship.BLUP” function in the R package rrBLUP version 4.6.1 (Endelman, 2011), while Bayesian linear regression models (BRR, BL, and BayesB) were executed using the R package BGLR ver. 1.0.363. Prediction accuracies (*r*) were estimated using 500 replicates of ten‐fold cross‐validation, with *r* computed as the mean Pearson's correlation coefficient between the genomic estimated breeding value (GEBV) and the observed health status.

To assess the effectiveness of GWAS‐associated SNPs and validate their potential, we considered two genomic prediction scenarios. Firstly, cross‐validation was conducted on dataset A with a training and testing population ratio of 60/40, involving subsets of 100, 200, 500, 1000, 2500, 5000, and 10,000 SNPs selected based on the most significant GWAS results. These results were then compared to sets of randomly selected SNPs from the genome, with each set having an equal number of SNPs as the original set being tested. Missing SNP data were imputed using the “A.mat” function from the R package rrBLUP version 4.6. (Endelman, 2011). The “mixed.solve” function in rrBLUP ver. 4.6.1 (Endelman, 2011) was employed with 500 iterations of ten‐fold cross‐validation. Prediction accuracy was determined as the mean Pearson correlation between GEBV and health status across the 500 replications. Individuals within the validation population were categorized into high‐ and low‐ susceptibility groups based on their GEBV. Healthy individuals were assigned GEBV values above the median, while diseased individuals were assigned values below the median. The accuracy of this classification was assessed using the formula: *f* = correct assignments/total assignments, where correct assignments referred to those that aligned with the observed phenotypes.

In the second scenario, genomic prediction models were trained on dataset A and tested on dataset B. The same subsets of SNPs from the previous scenario with the most significant GWAS results and randomly selected SNPs were used for the training and testing populations. To enhance the robustness and reliability of the analysis, the random selection of SNP datasets was repeated 10 times. It's noteworthy that a successful genotyping rate of over 90% was achieved for SNPs from dataset A in dataset B. The health status vector for each individual, denoted as y, was predicted using the rrBLUP model as: *y* = *Xβ* + *ε*, where *β* represents a vector of the marker effects and Var[*ε*] is the residual variance. The design matrix X contains the genotype information, where each row represents an individual and each column represents an SNP. Missing marker data were imputed using the “A.mat” function from the R package rrBLUP version 4.6.1 (Endelman, 2011). The effect sizes of each considered SNP (β) were estimated using the “mixed.solve” function in the rrBLUP ver. 4.6.1 package (Endelman, 2011). Genomic estimated breeding values (GEBV) for each tree in the validation population (dataset B) were calculated using the effect sizes obtained from dataset A. GEBVs were computed as the sum of the additive genetic effects of the SNPs found in dataset B. Trees within the validation population were categorized into high and low susceptibility groups as described above. The relationships between the GEBVs and the tree phenotypes (health status, defoliation, vitality, trunk condition, Syn, and SynT) were calculated using the Pearson correlation coefficient. In addition, for the random sets, prediction accuracy was determined as the mean Pearson correlation between GEBV and the health status indicators across the 10 sets for each size of SNP.

## 3 RESULTS

### 3.1 Sequencing and genotyping data

Raw data and mapping statistics are provided in Table S2. Approximately 1.99 and 178 million raw sequence reads per individual were generated for datasets A and B, respectively. Over 97% of reads for both datasets were mapped to the *F. excelsior* genome assembly.

After mapping the reads to the reference genome, over 2.5 million SNPs were detected in dataset A, while dataset B revealed 56 million SNPs. The SNP loci identified in this study were distributed on all 23 chromosomes (Table S3). However, after filtering, 28,817 loci for dataset A and 7,882,379 for dataset B remained, representing high‐confidence SNPs.

The frequency of unfiltered SNPs was 3.28 SNPs per 1 kilobase pair (kbp) for dataset A, and 70.98 SNPs/kbp for dataset B. After filtering, the frequency of SNPs was reduced to 0.04 and 10.50 SNPs/kbp for datasets A and B, respectively (Table S3). In dataset A, chromosome 1 had the highest number of unfiltered SNPs (181,986). However, for filtered SNPs, chromosome 2 had the highest number of SNPs (2022). In dataset B, chromosome 1 had the most SNPs for both unfiltered (3,733,633) and filtered (521,245) SNPs.

For dataset A, the average percentage of missing data per individual was 8.31%. Due to the high missing data ratio, eight individuals were removed from further analyses. For dataset B, the average missing data ratio per individual was 3.68%.

### 3.2 Genetic structure

The final dataset of SNPs from dataset A was compared to dataset B, and it was found that 26,757 out of 28,817 SNPs from dataset A were variable within dataset B. After applying additional filtering based on a MAF >0.05, a total of 26,134 SNPs were retained for the analysis of the genetic structure of all specimens analyzed in both datasets.

**FIGURE 2:**
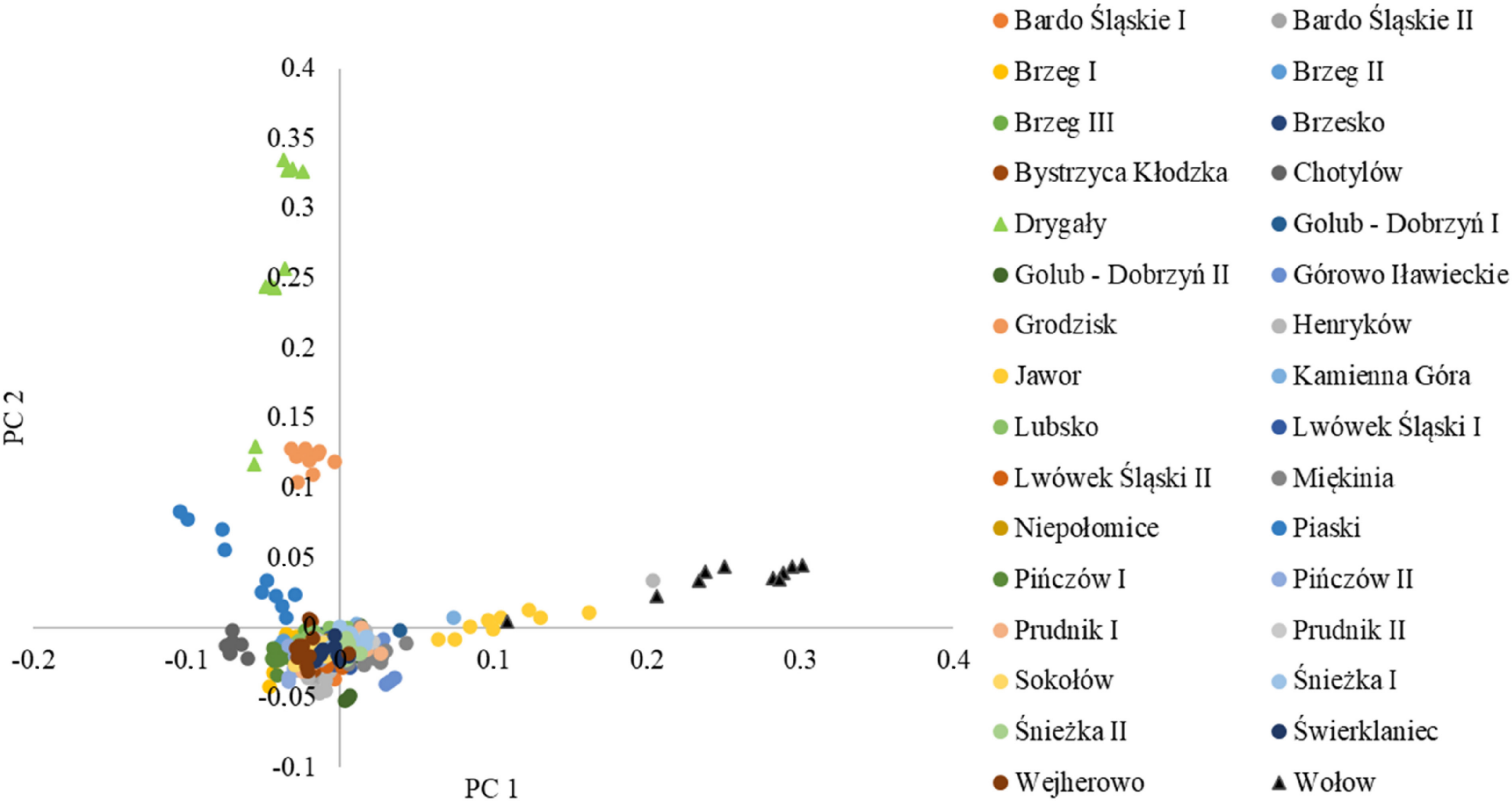
Principal component analysis plot of PC1 and PC2, based on 26,757 SNPs scored in 32 populations of common ash. The populations considered as outliers are marked with a triangle.

The variances explained by the first two PCA components were 0.0365 and 0.0294, respectively, indicating no clear genetic structure (Figure 2). The individuals form one large group, with outliers from the Drygały and Wołów populations. However, the fastSTRUCTURE function “chooseK” indicated *K* = 2 as the optimal number of clusters. The graphical representation of the membership coefficients per individual to the clusters is presented in Figure S1. The majority of individuals in the analyzed dataset were assigned to the first group (blue), while individuals from the Drygały, Jawor, and Wołów populations, which were outliers in PCA, were classified as belonging to the second group (orange).

### 3.3 GWAS

**FIGURE 3:**
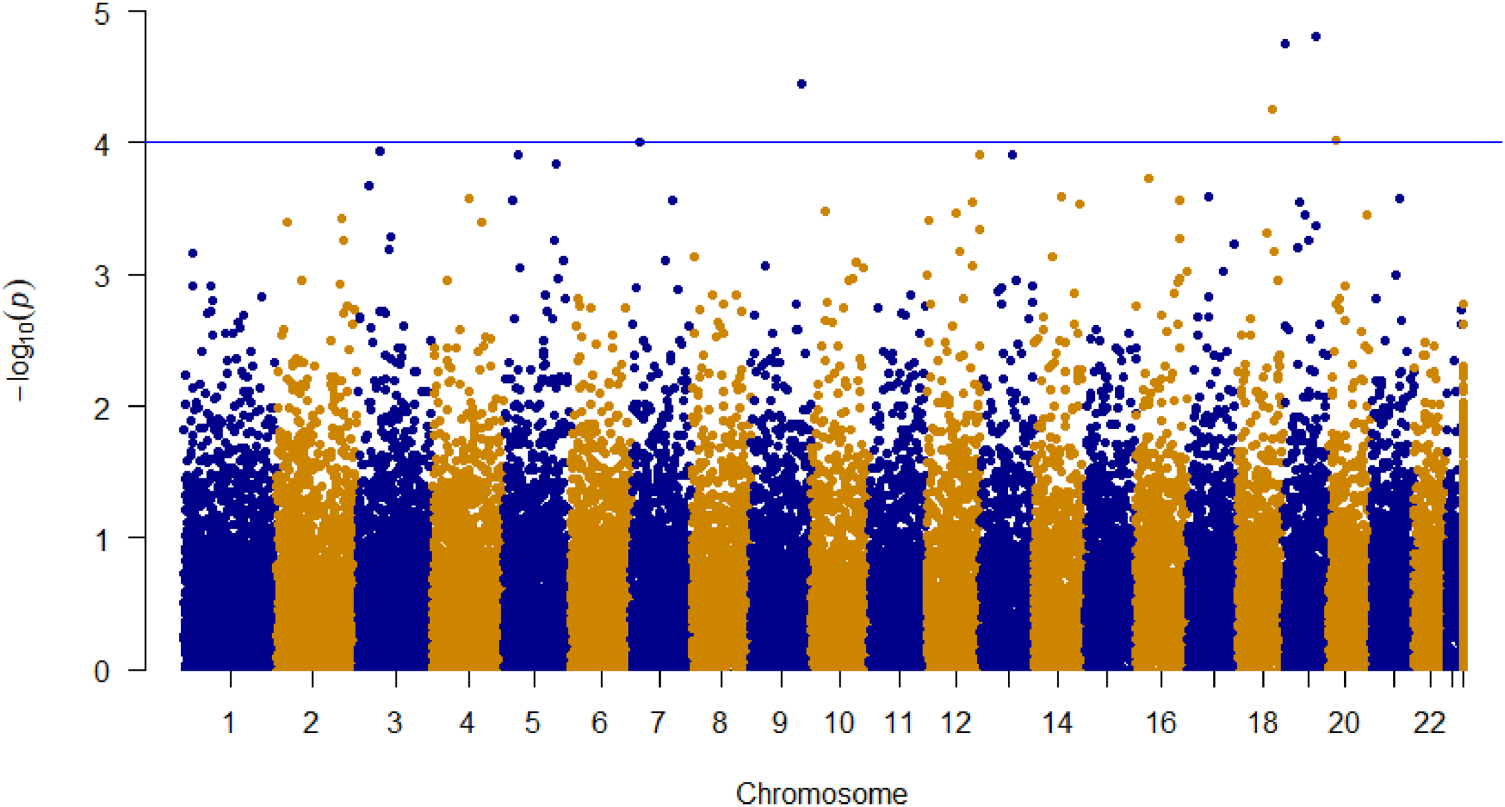
Manhattan plot of the results from the GWAS for health status in dataset A. Blue line indicates the threshold of the *p*‐value ≤1 × 10^−4^.

The GWAS analysis detected six SNP loci, showing a significant association with health status in dataset A, with the *p*‐values ranging from 1.55 × 10^−5^ to 9.79 × 10^−5^ (Figure 3, Table S4). However, it is important to note that these markers are situated in non‐coding regions of the genome. While a relatively lenient threshold was applied, it is essential to emphasize that the primary aim of these findings is to nominate loci for subsequent genomic prediction analyses.

### 3.4 Genomic prediction

**FIGURE 4:**
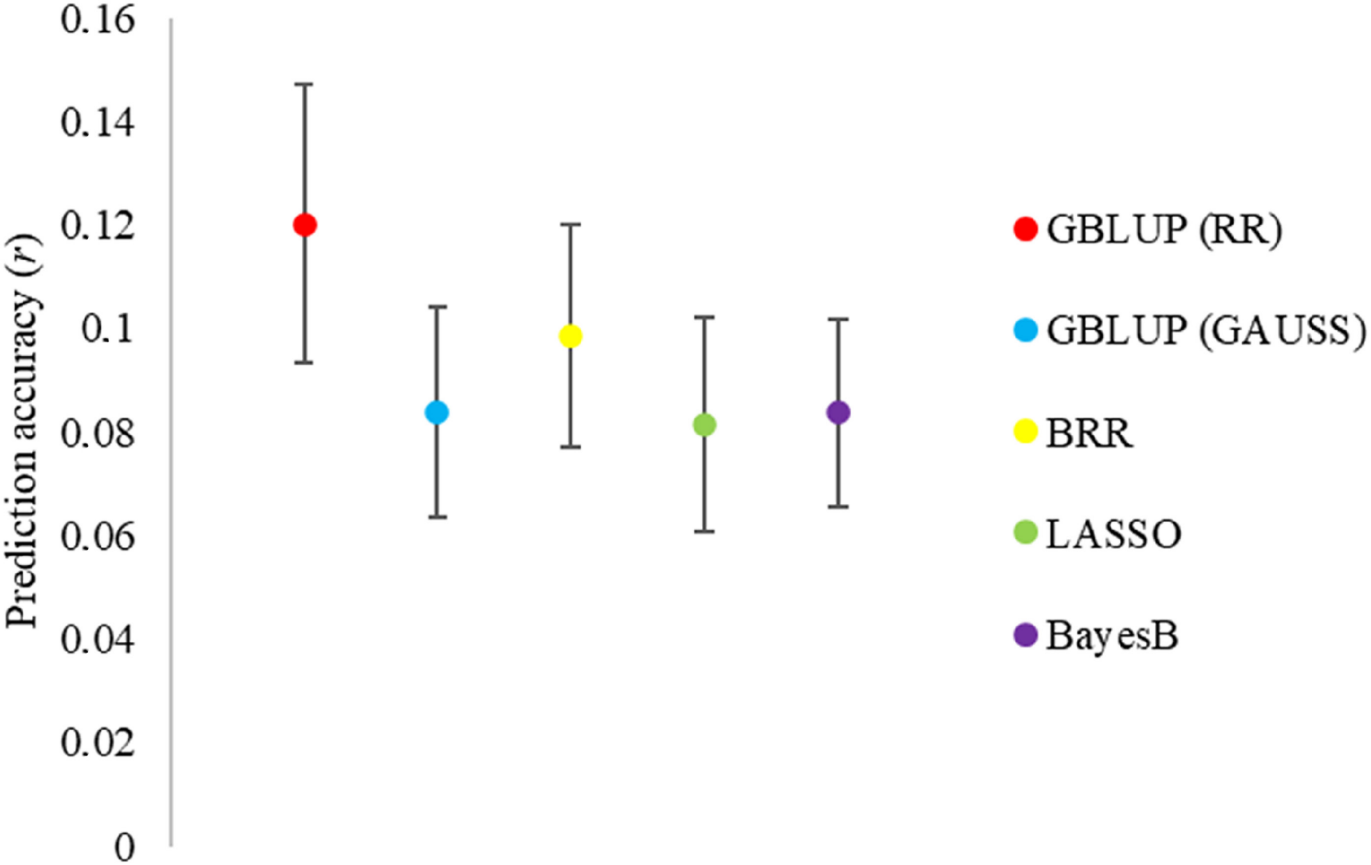
Genomic prediction accuracy for five predictive models (GBLUP (RR), GBLUP (GAUSS), BRR, LASSO, BayesB) using 28,817 SNPs to predict the health status of individuals in dataset A. Error bars represent the means ± standard error (based on 500 replicates of ten‐fold cross‐validation).

We evaluated the accuracy of the GP model for predicting the health status of individuals in dataset A using all 28,817 SNPs and five regression models previously described. The ten‐fold cross‐validations (CVs) showed *r* values ranging from 0.082 to 0.120 (Figure 4), but none of them were statistically significant. Among the models, GBLUP (RR) demonstrated superiority (*r* = 0.12; *p*‐value = 0.027) in predicting the health status of individuals in dataset A (Figure 4), and this was used for further analysis.

**FIGURE 5:**
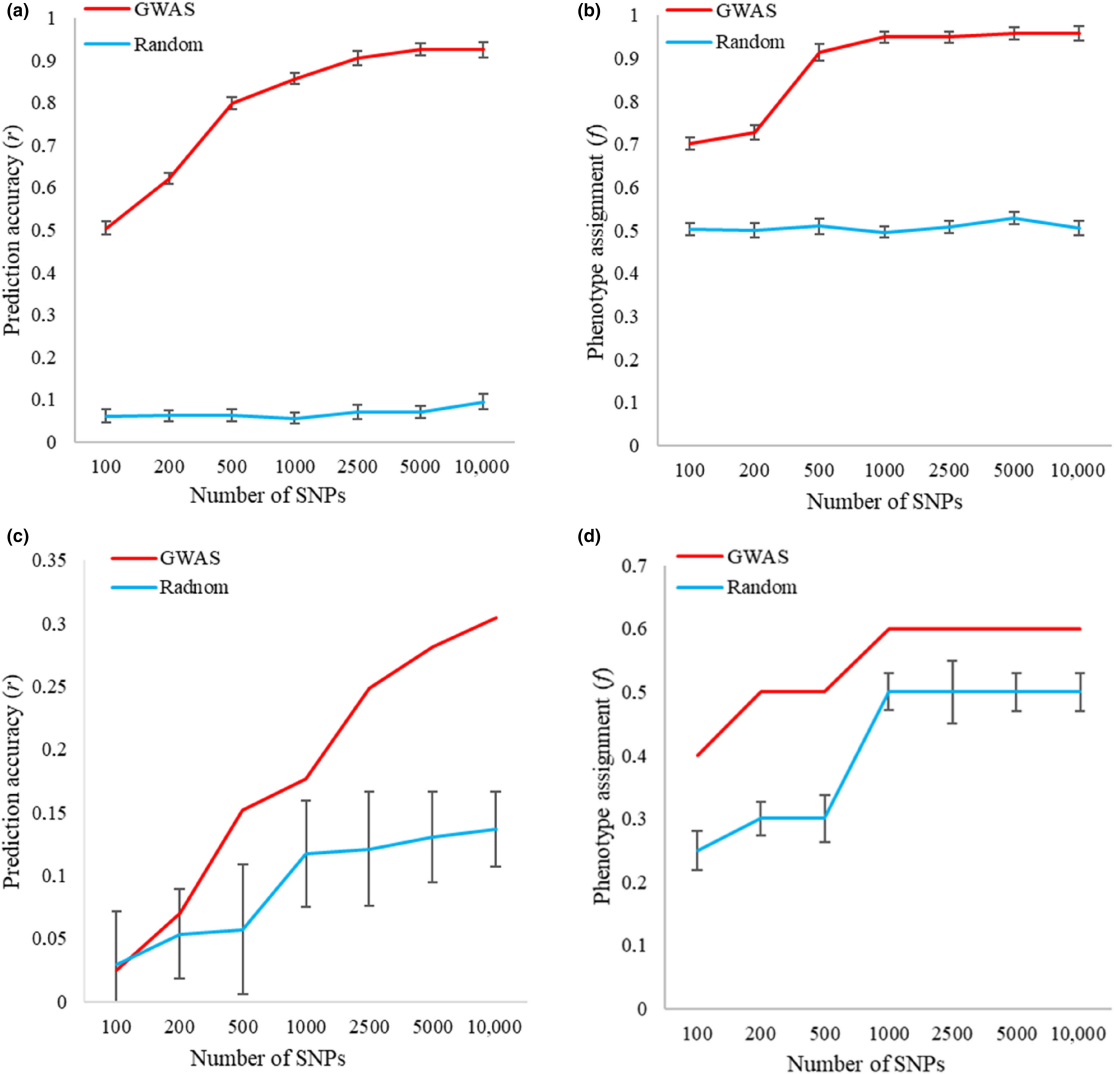
Genomic prediction accuracy and phenotype assignment results using the cross‐validation model conducted on dataset A (a, b) and training the model on dataset A and testing its performance on dataset B (c, d) to predict health status of common ash. In panels (a, b), error bars represent the means ± standard error (based on 500 replicates of ten‐fold cross‐validation). In panels (c, d), error bars for random datasets represent the means ± standard error (based on the selection of random SNP datasets performed 10 times).

The results of cross‐validation conducted on dataset A using various SNP datasets are presented in Figure 5a,b. The genomic prediction accuracy (Figure 5a) was significantly high and improved as the number of SNPs from the GWAS datasets increased. For the 2500 SNP dataset, the prediction accuracy reached *r* = 0.91 (*p*‐value = 2.2 × 10^−16^) and continued to increase, reaching *r* = 0.92 (*p*‐value = 2.2 × 10^−16^) in the 10,000 SNP dataset. Furthermore, accurate prediction of tree health status (phenotype assignment) exceeded 0.9 across SNP sets ranging from 500 to 10,000 SNPs (Figure 5b).

In contrast, no significant results were obtained when training the model on dataset A and testing its performance on dataset B (Figure 5b,c). Prediction accuracy increased with the number of SNPs, reaching a maximum value of *r* = 0.30 (*p*‐value = 0.16) for the 10,000 SNP dataset (Figure 5c). However, the correct assignment of health status was only 0.6 for datasets ranging from 1000 to 10,000 SNPs (Figure 5d). It is worth noting, that this was only slightly higher than the results achieved using randomly selected SNP sets of the same size. The estimated effect sizes for SNPs in sets of sizes 100, 200, 500, 1000, 2500, 5000, and 10,000 obtained in cross‐validation in dataset A are shown in Tables S5–S11.

The results showing the correlation of GEBV in dataset B with the remaining health status indicators (defoliation, vitality, Syn, trunk condition, and SynT) are presented in Figure S2. Generally, prediction accuracy was higher when utilizing the top SNPs selected by GWAS compared to randomly selected SNPs, particularly with larger SNP numbers (Figure S2). However, there were exceptions for trunk condition and the SynT index, where prediction accuracy was higher for sets with a smaller number of loci. Notably, significant prediction accuracy was observed with the top 2500 SNPs (*r* = 0.45; *p*‐value = 0.045), 5000 SNPs (*r* = 0.48; *p*‐value = 0.033), and 10,000 SNPs (*r* = 0.51; *p*‐value = 0.027) for defoliation. A similar pattern was visible for Syn, although the genomic prediction indicator was not statistically significant. Furthermore, significantly high prediction accuracy was achieved with 100 SNPs (*r* = 0.60; *p*‐value = 0.006) and 200 SNPs (*r* = 0.62; *p*‐value = 0.004). However, this pattern contradicts theoretical predictions and introduces uncertainty into our analysis. Therefore, we abstain from interpretation and acknowledge the necessity for further investigation to accurately elucidate its implications. The estimated effect sizes for SNPs in sets of sizes 2500, 5000, and 10,000 obtained in training the whole dataset A are provided in Tables S12–S14.

## 4 DISCUSSION

The increasing occurrence of introduced insect pests and pathogens due to anthropogenic environmental changes poses a significant threat to tree species worldwide, including common ash (*Fraxinus excelsior* L.). Previous genetic studies have demonstrated the heritability and polygenic nature of resistance to *Hymenoscyphus fraxineus* in common ash, suggesting that these trees may respond well to genomic selection, enabling the breeding or evolution of durable increased resistance. Therefore, GWAS and GP appear to be the most suitable tools for studying and identifying genetic markers that are associated with this complex trait. In this study, we assessed the capability of GWAS and GP to identify potential genetic markers that are associated with resistance to ash dieback.

### 4.1 Genetic structure

The genetic structure analysis based on PCA revealed that the first two principal components explained a relatively low amount of variance (PC1: 0.0365; PC2: 0.0294), suggesting that there is no clear genetic structure among the individuals (Figure 2). The relatively low differentiation between common ash populations at the nuclear genome may be attributed to pollen‐mediated homogenizing gene flow (Heuertz, Fineschi, et al., 2004; Heuertz, Hausman, et al., 2004). However, further examination using the fastSTRUCTURE software showed that *K* = 2 was the optimal number of clusters, indicating the presence of two distinct groups within the analyzed dataset. The observed genetic differentiation between the second group of populations (Drygały, Jawor and Wołów) and the rest of the individuals may reflect the postglacial colonization of Poland from the refugia located in the eastern Alps and the Balkans. It is expected that the southwestern populations of ash originate from the refugium in the eastern Alps, while the northeastern populations originate from the Balkan refugium (Heuertz, Fineschi, et al., 2004; Heuertz, Hausman, et al., 2004). These findings emphasize the significance of considering local genetic diversity and structure when formulating strategies for resistance breeding and conservation initiatives in common ash populations.

### 4.2 Genetic association

The GWAS analysis allowed us to identify six SNP loci strongly associated (p‐value ≤1 × 10^−4^) with the health status of common ash, as depicted in Figure 3 and listed in Table S4. However, the existing genetic structure of the common ash population can lead to false‐positive associations in GWAS analysis (Korte & Farlow, 2013). To address this issue, we applied a correction method by incorporating the first four principal components (PCs) along with a relative kinship matrix (*K*) in our GWAS analysis. This approach allowed us to account for the population structure and relatedness among individuals, reducing the risk of spurious associations and increasing the accuracy of our genetic association findings.

Previous research using associative transcriptomics has also identified a small number of immune‐related genes associated with increased resistance to *H. fraxineus* in Danish resistance trials. This includes one SNP based on cDNA and two gene expression markers (Harper et al., 2016). Further analysis of this data revealed an additional 20 gene expression markers associated with disease symptoms (Sollars et al., 2017), although none of the genes identified in this study corresponded to those previously found in immune‐related genes. Furthermore, the comprehensive sequencing of 1250 trees and a subsequent genome‐wide association study led to the identification of an impressive 3149 SNPs that were significantly associated with disease resistance (Stocks et al., 2019). Intriguingly, out of the 192 most significant SNPs, 61 were associated with genes that have homologs that are known to be involved in plant–pathogen interactions (Stocks et al., 2019).

The low number of genes related to ash dieback found in this study could be attributed to several factors. One possible reason is the use of ddRAD data, which represents only a small fraction of the genome, making it impossible to identify all the genes. Another possible reason is the complexity of the trait being studied. Disease resistance is a multifaceted trait influenced by various genetic and environmental factors, making it challenging to identify all the genes involved. Moreover, the sample size and statistical power of the GWAS could influence the number of identified genes. With a small sample size, the study may lack sufficient statistical power to detect all of the relevant genetic associations, leading to a lower number of genes being identified. Furthermore, the presence of population‐specific adaptations and environmental interactions may additionally complicate the identification of genes associated with disease resistance.

### 4.3 Genomic prediction

Genomic selection presents a promising avenue for enhancing disease resistance in ash trees within breeding programs. It is widely recognized that genomic prediction accuracy tends to increase with higher marker density (Elshire et al., 2011; Zhang et al., 2019). However, considerations regarding the cost of genotyping and phenotyping are paramount, as they can significantly impact the feasibility of large‐scale implementation. In our study, we opted for ddRAD data for genotyping, a cost‐effective and efficient method for identifying SNPs across the genome. Additionally, we adopted a genomic selection strategy where a relatively low number of SNPs selected by GWAS can replace a high density of SNPs without compromising prediction accuracy.

Among the five genomic prediction methods tested, GBLUP (RR) exhibited higher prediction accuracy compared to other models in predicting the health status of individuals in dataset A. However, it's important to note that the difference in accuracy was not statistically significant. Nonetheless, this underscores the potential of the GBLUP (RR) model for accurate phenotypic prediction, aligning with its recognized robust performance under low marker density conditions (Habier et al., 2007). Furthermore, our findings align with earlier studies in maize (Gowda et al., 2015; Zhao et al., 2012), further reinforcing the credibility and applicability of the GBLUP (RR) model across different plant species.

Both genomic prediction scenarios applied in this study demonstrated that utilizing the top‐ranked SNPs identified through GWAS yielded higher prediction accuracies for disease resistance traits compared to randomly selected SNPs. This suggests that the loci influencing resistance to ash dieback, as identified through GWAS, played a significant role in enhancing prediction accuracies, even with low‐density SNP panels. By pinpointing these key genetic markers associated with disease resistance, GWAS provides valuable insights for targeted breeding programs aimed at improving ash tree resilience to ash dieback. Previous studies across various plant species have also highlighted the effectiveness of leveraging GWAS information to improve genomic selection strategies (Spindel et al., 2016; Stocks et al., 2019; Zhang et al., 2014). Our findings further underscore the importance of integrating GWAS findings into genomic prediction models to enhance breeding efforts for disease resistance in trees.

The levels of prediction accuracy in our study are relatively high and are comparable to those observed in genomic selection studies of other plant species (Biazzi et al., 2017; Grinberg et al., 2016; Müller et al., 2017; Resende, Resende et al., 2012; Resende, Muñoz et al., 2012; Slavov et al., 2014; Spindel et al., 2016; Stocks et al., 2019; Yamashita et al., 2020). Cross‐validation analyses using dataset A demonstrated high genomic prediction accuracy, with correlation coefficients (*r*) surpassing 0.9 for datasets ranging from 2500 to 10,000 SNPs. Moreover, predictions of tree health status (*f*) achieved an accuracy exceeding 0.9 across SNP sets ranging from 500 to 10,000 SNPs from the GWAS datasets. However, when the model trained on dataset A was tested on dataset B, no significant results were observed for health status prediction. The maximum prediction accuracy reached a value of *r* = 0.30 (*p*‐value = 0.16) for the 10,000 SNP dataset, while the correct assignment of health status achieved an accuracy of *f* = 0.6 for datasets ranging from 1000 to 10,000 SNPs. In genomic prediction scenarios where populations from the test set are not included in model training, prediction accuracy may decrease due to genetic and environmental differences between populations, influencing the model's effectiveness in predicting phenotypic traits.

In contrast, significant statistical results were obtained in a study conducted by Stocks et al. (2019) where genomic prediction was used to determine the health status of common ash in southeast England. They demonstrated significant predictions of ash tree health using pool‐seq data from 1250 trees for training and testing on 150 individuals, which were not part of the DNA pools. In this scenario, they achieved the highest accuracy with a correlation coefficient (*r*) of observed scores and GEBV of *r* = 0.35, along with a frequency of correct allocations of *f* = 0.67.

The additional correlations of GEBV in dataset B with other health status indicators revealed notable prediction accuracies for defoliation across specific SNP sets: 2500 SNPs (*r* = 0.45; *p*‐value = 0.045), 5000 SNPs (*r* = 0.48; *p*‐value = 0.033), and 10,000 SNPs (*r* = 0.51; *p*‐value = 0.027), selected based on GWAS findings. This suggests that defoliation may serve as a superior indicator of tree health, possibly more directly linked to the tree's response to fungal infection causing ash dieback.

A common limitation of genomic prediction models is their high population specificity (Müller et al., 2017; Wientjes et al., 2013). Hence, our results hold the potential for expediting the breeding of ash dieback‐resistant trees in southwestern Poland (the region covered by dataset A). Unfortunately, widespread alleles involved in ash dieback resistance across all analyzed populations in Poland were not identified. Notably, in the second genomic prediction scenario, only 20 samples were used for testing, potentially limiting the scope and generalizability of our findings. Further research with larger sample sizes and diverse populations is crucial to fully elucidate the genetic basis of ash dieback resistance across different regions in Poland. Moreover, leveraging whole‐genome sequencing can lead to a higher marker density, facilitating the discovery of additional genetic variants associated with disease resistance.

## 5 CONCLUSIONS

The present study demonstrates the potential of a genomic selection strategy for enhancing disease resistance in ash trees within breeding programs. We found that utilizing a relatively low number of SNPs selected through genome‐wide association studies (GWAS) can yield high prediction accuracies, highlighting the efficiency of this approach. Cross‐validation analyses showcased high genomic prediction accuracy, predicting tree health status with over 90% accuracy across the top SNP sets ranging from 500 to 10,000 SNPs from the GWAS datasets. Our findings illuminate the potential for expediting the breeding of ash dieback‐resistant trees in southwestern Poland. To advance the promising prospects of genomic selection, future endeavors should prioritize the expansion of training populations and the implementation of whole‐genome sequencing. These steps hold the potential for further bolstering the effectiveness of genomic selection, ultimately contributing to the preservation and fortification of common ash populations in the face of pressing threats, such as ash dieback.

## CONFLICT OF INTEREST STATEMENT

The authors declare no conflict of interest.

## Supporting information


Tables S1–S14.

Figures S1–S2.

## Data Availability

Double digest restriction‐site associated DNA (ddRAD) sequencing data for dataset A are publicly available at the NCBI BioProject PRJNA1063327 (https://www.ncbi.nlm.nih.gov/bioproject/1063327). Whole genome sequencing data for dataset B are publicly available at the NCBI BioProject PRJNA1062570 (https://www.ncbi.nlm.nih.gov/bioproject/?term=PRJNA1062570).

## References

[eva13694-bib-0001] Alfaro, R. I. , King, J. N. , & vanAkker, L. (2013). Delivering Sitka spruce with resistance against white pine weevil in British Columbia, Canada. The Forestry Chronicle, 89, 235–245. 10.5558/tfc2013-042

[eva13694-bib-0002] Andrews, S. (2010). FASTQC. A quality control tool for high throughput sequence data.

[eva13694-bib-0003] Baral, H.‐O. , Queloz, V. , & Hosoya, T. (2014). *Hymenoscyphus fraxineus*, the correct scientific name for the fungus causing ash dieback in Europe. IMA Fungus, 5(1), 79–80. 10.5598/imafungus.2014.05.01.09 25083409 PMC4107900

[eva13694-bib-0004] Bartnik, C. , Michalcewicz, J. , & Ciach, M. (2015). Dutch elm disease and the habitat of endangered Rosalia longicorn *Rosalia alpina* (L.): A conservation paradox? Polish Journal of Ecology, 63(3), 440–447, 448. 10.3161/15052249PJE2015.63.3.013

[eva13694-bib-0005] Beaulieu, J. , Nadeau, S. , Ding, C. , Celedon, J. M. , Azaiez, A. , Ritland, C. , Laverdière, J. P. , Deslauriers, M. , Adams, G. , Fullarton, M. , Bohlmann, J. , Lenz, P. , & Bousquet, J. (2020). Genomic selection for resistance to spruce budworm in white spruce and relationships with growth and wood quality traits. Evolutionary Applications, 13(10), 2704–2722. 10.1111/eva.13076 33294018 PMC7691460

[eva13694-bib-0006] Biazzi, E. , Nazzicari, N. , Pecetti, L. , Brummer, E. C. , Palmonari, A. , Tava, A. , & Annicchiarico, P. (2017). Genome‐wide association mapping and genomic selection for alfalfa (*Medicago sativa*) forage quality traits. PLoS One, 12(1), e0169234. 10.1371/journal.pone.0169234 28068350 PMC5222375

[eva13694-bib-0007] Bolger, A. M. , Lohse, M. , & Usadel, B. (2014). Trimmomatic: A flexible trimmer for Illumina sequence data. Bioinformatics, 30(15), 2114–2120. 10.1093/bioinformatics/btu170 24695404 PMC4103590

[eva13694-bib-0008] Bouvet, J.‐M. , Makouanzi Ekomono, C. G. , Brendel, O. , Laclau, J.‐P. , Bouillet, J.‐P. , & Epron, D. (2020). Selecting for water use efficiency, wood chemical traits and biomass with genomic selection in a eucalyptus breeding program. Forest Ecology and Management, 465, 118092. 10.1016/j.foreco.2020.118092

[eva13694-bib-0009] Boyd, I. L. , Freer‐Smith, P. H. , Gilligan, C. A. , & Godfray, H. C. J. (2013). The consequence of tree pests and diseases for ecosystem services. Science, 342(6160), 1235773. 10.1126/science.1235773 24233727

[eva13694-bib-0010] Cappa, E. P. , Chen, C. , Klutsch, J. G. , Sebastian‐Azcona, J. , Ratcliffe, B. , Wei, X. , da Ros, L. , Ullah, A. , Liu, Y. , Benowicz, A. , Sadoway, S. , Mansfield, S. D. , Erbilgin, N. , Thomas, B. R. , & el‐Kassaby, Y. A. (2022). Multiple‐trait analyses improved the accuracy of genomic prediction and the power of genome‐wide association of productivity and climate change‐adaptive traits in lodgepole pine. BMC Genomics, 23(1), 536. 10.1186/s12864-022-08747-7 35870886 PMC9308220

[eva13694-bib-0011] Chaudhary, R. , Rönneburg, T. , Stein Åslund, M. , Lundén, K. , Durling, M. B. , Ihrmark, K. , & Stenlid, J. (2020). Marker‐trait associations for tolerance to ash dieback in common ash (*Fraxinus excelsior* L.). Forests, 11(10), 1083.

[eva13694-bib-0012] Coker, T. L. R. , Rozsypálek, J. , Edwards, A. , Harwood, T. P. , Butfoy, L. , & Buggs, R. J. A. (2019). Estimating mortality rates of European ash (*Fraxinus excelsior*) under the ash dieback (*Hymenoscyphus fraxineus*) epidemic. Plants, People, Planet, 1(1), 48–58. 10.1002/ppp3.11

[eva13694-bib-0013] Danecek, P. , Auton, A. , Abecasis, G. , Albers, C. A. , Banks, E. , DePristo, M. A. , Handsaker, R. E. , Lunter, G. , Marth, G. T. , Sherry, S. T. , McVean, G. , Durbin, R. , & 1000 Genomes Project Analysis Group . (2011). The variant call format and VCFtools. Bioinformatics, 27(15), 2156–2158. 10.1093/bioinformatics/btr330 21653522 PMC3137218

[eva13694-bib-0014] Del Fabbro, C. , Scalabrin, S. , Morgante, M. , & Giorgi, F. M. (2013). An extensive evaluation of read trimming effects on Illumina NGS data analysis. PLoS One, 8(12), e85024. 10.1371/journal.pone.0085024 24376861 PMC3871669

[eva13694-bib-0015] DePristo, M. A. , Banks, E. , Poplin, R. , Garimella, K. V. , Maguire, J. R. , Hartl, C. , Philippakis, A. A. , del Angel, G. , Rivas, M. A. , Hanna, M. , McKenna, A. , Fennell, T. J. , Kernytsky, A. M. , Sivachenko, A. Y. , Cibulskis, K. , Gabriel, S. B. , Altshuler, D. , & Daly, M. J. (2011). A framework for variation discovery and genotyping using next‐generation DNA sequencing data. Nature Genetics, 43(5), 491–498. 10.1038/ng.806 21478889 PMC3083463

[eva13694-bib-0016] Dmyterko, E. , & Bruchwald, A. (1998). Weryfikacja metod określania uszkodzenia drzewostanów dębowych. Sylwan, 142(12), 11–21.

[eva13694-bib-0017] Dobrowolska, D. , Hein, S. , Oosterbaan, A. , Wagner, S. , Clark, J. , & Skovsgaard, J. P. (2011). A review of European ash (*Fraxinus excelsior* L.): Implications for silviculture. Forestry: An International Journal of Forest Research, 84(2), 133–148. 10.1093/forestry/cpr001

[eva13694-bib-0018] Dobson, A. (2009). Climate variability, global change, immunity, and the dynamics of infectious diseases. Ecology, 90(4), 920–927. 10.1890/08-0736.1 19449686

[eva13694-bib-0019] Ellison, A. M. , Bank, M. S. , Clinton, B. D. , Colburn, E. A. , Elliott, K. , Ford, C. R. , & Webster, J. R. (2005). Loss of foundation species: Consequences for the structure and dynamics of forested ecosystems. Frontiers in Ecology and the Environment, 3(9), 479–486. 10.1890/1540-9295(2005)003[0479:LOFSCF]2.0.CO;2

[eva13694-bib-0020] Elshire, R. J. , Glaubitz, J. C. , Sun, Q. , Poland, J. A. , Kawamoto, K. , Buckler, E. S. , & Mitchell, S. E. (2011). A robust, simple genotyping‐by‐sequencing (GBS) approach for high diversity species. PLoS One, 6(5), e19379. 10.1371/journal.pone.0019379 21573248 PMC3087801

[eva13694-bib-0021] Endelman, J. B. (2011). Ridge regression and other kernels for genomic selection with R package rrBLUP. The Plant Genome, 4(3), 250–255. 10.3835/plantgenome2011.08.0024

[eva13694-bib-0022] Enderle, R. , Nakou, A. , Thomas, K. , & Metzler, B. (2015). Susceptibility of autochthonous German Fraxinus excelsior clones to *Hymenoscyphus pseudoalbidus* is genetically determined. Annals of Forest Science, 72(2), 183–193. 10.1007/s13595-014-0413-1

[eva13694-bib-0023] Fisher, M. C. , Henk, D. A. , Briggs, C. J. , Brownstein, J. S. , Madoff, L. C. , McCraw, S. L. , & Gurr, S. J. (2012). Emerging fungal threats to animal, plant and ecosystem health. Nature, 484(7393), 186–194. 10.1038/nature10947 22498624 PMC3821985

[eva13694-bib-0024] Gowda, M. , das, B. , Makumbi, D. , Babu, R. , Semagn, K. , Mahuku, G. , Olsen, M. S. , Bright, J. M. , Beyene, Y. , & Prasanna, B. M. (2015). Genome‐wide association and genomic prediction of resistance to maize lethal necrosis disease in tropical maize germplasm. Theoretical and Applied Genetics, 128(10), 1957–1968. 10.1007/s00122-015-2559-0 26152570 PMC4572053

[eva13694-bib-0025] Grattapaglia, D. , & Resende, M. D. V. (2011). Genomic selection in forest tree breeding. Tree Genetics & Genomes, 7(2), 241–255. 10.1007/s11295-010-0328-4

[eva13694-bib-0026] Grinberg, N. F. , Lovatt, A. , Hegarty, M. , Lovatt, A. , Skøt, K. P. , Kelly, R. , Blackmore, T. , Thorogood, D. , King, R. D. , Armstead, I. , Powell, W. , & Skøt, L. (2016). Implementation of genomic prediction in *Lolium perenne* (L.) breeding populations. Frontiers in Plant Science, 7, 133. 10.3389/fpls.2016.00133 26904088 PMC4751346

[eva13694-bib-0027] Guo, C. , Yu, J. , Ho, T. Y. , Wang, L. , Song, S. , Kong, L. , & Liu, H. (2012). Dynamics of phytoplankton community structure in the South China Sea in response to the east Asian aerosol input. Biogeosciences, 9(4), 1519–1536. 10.5194/bg-9-1519-2012

[eva13694-bib-0028] Habier, D. , Fernando, R. L. , & Dekkers, J. C. M. (2007). The impact of genetic relationship information on genome‐assisted breeding values. Genetics, 177(4), 2389–2397. 10.1534/genetics.107.081190 18073436 PMC2219482

[eva13694-bib-0029] Harper, A. L. , McKinney, L. V. , Nielsen, L. R. , Havlickova, L. , Li, Y. , Trick, M. , Fraser, F. , Wang, L. , Fellgett, A. , Sollars, E. S. , Janacek, S. H. , Downie, J. A. , Buggs, R. J. , Kjær, E. D. , & Bancroft, I. (2016). Molecular markers for tolerance of European ash (*Fraxinus excelsior*) to dieback disease identified using associative transcriptomics. Scientific Reports, 6(1), 19335. 10.1038/srep19335 26757823 PMC4725942

[eva13694-bib-0030] Havrdová, L. , Novotná, K. , Zahradník, D. , Buriánek, V. , Pešková, V. , Šrůtka, P. , & Černý, K. (2016). Differences in susceptibility to ash dieback in Czech provenances of Fraxinus excelsior. Forest Pathology, 46(4), 281–288. 10.1111/efp.12265

[eva13694-bib-0031] Heffner, E. L. , Lorenz, A. J. , Jannink, J.‐L. , & Sorrells, M. E. (2010). Plant breeding with genomic selection: Gain per unit time and cost. Crop Science, 50(5), 1681–1690. 10.2135/cropsci2009.11.0662

[eva13694-bib-0032] Heuertz, M. , Fineschi, S. , Anzidei, M. , Pastorelli, R. , Salvini, D. , Paule, L. , Frascaria‐Lacoste, N. , Hardy, O. J. , Vekemans, X. , & Vendramin, G. G. (2004). Chloroplast DNA variation and postglacial recolonization of common ash (*Fraxinus excelsior* L.) in Europe. Molecular Ecology, 13(11), 3437–3452. 10.1111/j.1365-294X.2004.02333.x 15488002

[eva13694-bib-0033] Heuertz, M. , Hausman, J. F. , Hardy, O. J. , Vendramin, G. G. , Frascaria‐Lacoste, N. , & Vekemans, X. (2004). Nuclear microsatellites reveal contrasting patterns of genetic structure between western and southeastern European populations of the common ash (*Fraxinus excelsior* L.). Evolution, 58(5), 976–988. 10.1111/j.0014-3820.2004.tb00432.x 15212379

[eva13694-bib-0034] Hultberg, T. , Sandström, J. , Felton, A. , Öhman, K. , Rönnberg, J. , Witzell, J. , & Cleary, M. (2020). Ash dieback risks an extinction cascade. Biological Conservation, 244, 108516. 10.1016/j.biocon.2020.108516

[eva13694-bib-0035] Ingvarsson, P. K. , & Street, N. R. (2011). Association genetics of complex traits in plants. New Phytologist, 189(4), 909–922. 10.1111/j.1469-8137.2010.03593.x 21182529

[eva13694-bib-0036] Iwata, H. , Minamikawa, M. F. , Kajiya‐Kanegae, H. , Ishimori, M. , & Hayashi, T. (2016). Genomics‐assisted breeding in fruit trees. Breeding Science, 66(1), 100–115. 10.1270/jsbbs.66.100 27069395 PMC4780794

[eva13694-bib-0037] Jung, T. (2009). Beech decline in Central Europe driven by the interaction between phytophthora infections and climatic extremes. Forest Pathology, 39(2), 73–94. 10.1111/j.1439-0329.2008.00566.x

[eva13694-bib-0038] Kjær, E. D. , McKinney, L. V. , Nielsen, L. R. , Hansen, L. N. , & Hansen, J. K. (2012). Adaptive potential of ash (*Fraxinus excelsior*) populations against the novel emerging pathogen *Hymenoscyphus pseudoalbidus* . Evolutionary Applications, 5(3), 219–228. 10.1111/j.1752-4571.2011.00222.x 25568043 PMC3353348

[eva13694-bib-0039] Kopelman, N. M. , Mayzel, J. , Jakobsson, M. , Rosenberg, N. A. , & Mayrose, I. (2015). Clumpak: A program for identifying clustering modes and packaging population structure inferences across K. Molecular Ecology Resources, 15(5), 1179–1191. 10.1111/1755-0998.12387 25684545 PMC4534335

[eva13694-bib-0040] Korte, A. , & Farlow, A. (2013). The advantages and limitations of trait analysis with GWAS: A review. Plant Methods, 9(1), 29. 10.1186/1746-4811-9-29 23876160 PMC3750305

[eva13694-bib-0041] Kowalski, T. (2006). *Chalara fraxinea* sp. nov. associated with dieback of ash (*Fraxinus excelsior*) in Poland. Forest Pathology, 36(4), 264–270. 10.1111/j.1439-0329.2006.00453.x

[eva13694-bib-0042] Kruglyak, L. (2008). The road to genome‐wide association studies. Nature Reviews Genetics, 9(4), 314–318. 10.1038/nrg2316 18283274

[eva13694-bib-0043] Kumar, S. , Chagné, D. , Bink, M. C. , Volz, R. K. , Whitworth, C. , & Carlisle, C. (2012). Genomic selection for fruit quality traits in apple (malus × domestica Borkh.). PLoS One, 7(5), e36674. 10.1371/journal.pone.0036674 22574211 PMC3344927

[eva13694-bib-0044] Laverdière, J.‐P. , Lenz, P. , Nadeau, S. , Depardieu, C. , Isabel, N. , Perron, M. , Beaulieu, J. , & Bousquet, J. (2022). Breeding for adaptation to climate change: Genomic selection for drought response in a white spruce multi‐site polycross test. Evolutionary Applications, 15(3), 383–402. 10.1111/eva.13348 35386396 PMC8965362

[eva13694-bib-0045] Lenz, P. R. N. , Nadeau, S. , Mottet, M.‐J. , Perron, M. , Isabel, N. , Beaulieu, J. , & Bousquet, J. (2020). Multi‐trait genomic selection for weevil resistance, growth, and wood quality in Norway spruce. Evolutionary Applications, 13(1), 76–94. 10.1111/eva.12823 31892945 PMC6935592

[eva13694-bib-0046] Li, H. , & Durbin, R. (2009). Fast and accurate short read alignment with burrows–wheeler transform. Bioinformatics, 25(14), 1754–1760. 10.1093/bioinformatics/btp324 19451168 PMC2705234

[eva13694-bib-0047] Li, H. , Handsaker, B. , Wysoker, A. , Fennell, T. , Ruan, J. , Homer, N. , Marth, G. , Abecasis, G. , Durbin, R. , & 1000 Genome Project Data Processing Subgroup . (2009). The sequence alignment/map format and SAMtools. Bioinformatics, 25(16), 2078–2079. 10.1093/bioinformatics/btp352 19505943 PMC2723002

[eva13694-bib-0048] Li, J. , & Ji, L. (2005). Adjusting multiple testing in multilocus analyses using the eigenvalues of a correlation matrix. Heredity, 95(3), 221–227. 10.1038/sj.hdy.6800717 16077740

[eva13694-bib-0049] Lobo, A. , Hansen, J. K. , McKinney, L. V. , Nielsen, L. R. , & Kjær, E. D. (2014). Genetic variation in dieback resistance: Growth and survival of Fraxinus excelsior under the influence of *Hymenoscyphus pseudoalbidus* . Scandinavian Journal of Forest Research, 29(6), 519–526. 10.1080/02827581.2014.950603

[eva13694-bib-0050] Lobo, A. , McKinney, L. V. , Hansen, J. K. , Kjær, E. D. , & Nielsen, L. R. (2015). Genetic variation in dieback resistance in Fraxinus excelsior confirmed by progeny inoculation assay. Forest Pathology, 45(5), 379–387. 10.1111/efp.12179

[eva13694-bib-0051] Martín, J. A. , Domínguez, J. , Solla, A. , Brasier, C. M. , Webber, J. F. , Santini, A. , Martínez‐Arias, C. , Bernier, L. , & Gil, L. (2021). Complexities underlying the breeding and deployment of Dutch elm disease resistant elms. New Forests, 54, 661–696. 10.1007/s11056-021-09865-y 37361260 PMC10287581

[eva13694-bib-0052] Martín, J. A. , Sobrino‐Plata, J. , Rodríguez‐Calcerrada, J. , Collada, C. , & Gil, L. (2019). Breeding and scientific advances in the fight against Dutch elm disease: Will they allow the use of elms in forest restoration? New Forests, 50(2), 183–215. 10.1007/s11056-018-9640-x

[eva13694-bib-0053] Martin, M. (2011). Cutadapt removes adapter sequences from high‐throughput sequencing reads. EMBnet Journal, 17, 3. 10.14806/ej.17.1.200

[eva13694-bib-0054] McKinney, L. V. , Nielsen, L. R. , Collinge, D. B. , Thomsen, I. M. , Hansen, J. K. , & Kjær, E. D. (2014). The ash dieback crisis: Genetic variation in resistance can prove a long‐term solution. Plant Pathology, 63(3), 485–499. 10.1111/ppa.12196

[eva13694-bib-0055] McKinney, L. V. , Nielsen, L. R. , Hansen, J. K. , & Kjær, E. D. (2011). Presence of natural genetic resistance in *Fraxinus excelsior* (Oleraceae) to *Chalara fraxinea* (Ascomycota): An emerging infectious disease. Heredity (Edinb), 106(5), 788–797. 10.1038/hdy.2010.119 20823903 PMC3186218

[eva13694-bib-0056] Meger, J. , Kozioł, C. , Pałucka, M. , Burczyk, J. , & Chybicki, I. J. (2024). Genetic resources of common ash (*Fraxinus excelsior* L.) in Poland. BMC Plant Biology, 24(1), 1–15. 38481155 10.1186/s12870-024-04886-z PMC10935948

[eva13694-bib-0057] Menkis, A. , Bakys, R. , Stein Åslund, M. , Davydenko, K. , Elfstrand, M. , Stenlid, J. , & Vasaitis, R. (2020). Identifying *Fraxinus excelsior* tolerant to ash dieback: Visual field monitoring versus a molecular marker. Forest Pathology, 50(1), e12572. 10.1111/efp.12572

[eva13694-bib-0058] Meuwissen, T. H. E. , Hayes, B. J. , & Goddard, M. E. (2001). Prediction of Total genetic value using genome‐wide dense marker maps. Genetics, 157(4), 1819–1829. 10.1093/genetics/157.4.1819 11290733 PMC1461589

[eva13694-bib-0059] Muchero, W. , Sondreli, K. L. , Chen, J.‐G. , Urbanowicz, B. R. , Zhang, J. , Singan, V. , Yang, Y. , Brueggeman, R. S. , Franco‐Coronado, J. , Abraham, N. , Yang, J. Y. , Moremen, K. W. , Weisberg, A. J. , Chang, J. H. , Lindquist, E. , Barry, K. , Ranjan, P. , Jawdy, S. , Schmutz, J. , … LeBoldus, J. (2018). Association mapping, transcriptomics, and transient expression identify candidate genes mediating plant–pathogen interactions in a tree. Proceedings of the National Academy of Sciences, 115(45), 11573–11578. 10.1073/pnas.1804428115 PMC6233113 30337484

[eva13694-bib-0060] Mukrimin, M. , Kovalchuk, A. , Neves, L. G. , Jaber, E. H. A. , Haapanen, M. , Kirst, M. , & Asiegbu, F. O. (2018). Genome‐wide exon‐capture approach identifies genetic variants of Norway spruce genes associated with susceptibility to *Heterobasidion parviporum* infection. Frontiers in Plant Science, 9, 793. 10.3389/fpls.2018.00793 29946332 PMC6005875

[eva13694-bib-0061] Müller, B. S. F. , Neves, L. G. , de Almeida Filho, J. E. , Resende, M. F. R. , Muñoz, P. R. , dos Santos, P. , Filho, E. P. , Kirst, M. , & Grattapaglia, D. (2017). Genomic prediction in contrast to a genome‐wide association study in explaining heritable variation of complex growth traits in breeding populations of eucalyptus. BMC Genomics, 18(1), 524. 10.1186/s12864-017-3920-2 28693539 PMC5504793

[eva13694-bib-0062] Muñoz, F. , Marçais, B. , Dufour, J. , & Dowkiw, A. (2016). Rising out of the ashes: Additive genetic variation for crown and collar resistance to *Hymenoscyphus fraxineus* in Fraxinus excelsior. Phytopathology, 106(12), 1535–1543. 10.1094/phyto-11-15-0284-r 27349738

[eva13694-bib-0063] Neale, D. B. , & Kremer, A. (2011). Forest tree genomics: Growing resources and applications. Nature Reviews Genetics, 12(2), 111–122. 10.1038/nrg2931 21245829

[eva13694-bib-0064] Orlova‐Bienkowskaja, M. J. , & Volkovitsh, M. G. (2015). Range expansion of *Agrilus convexicollis* in European Russia expedited by the invasion of the emerald ash borer, *Agrilus planipennis* (coleoptera: Buprestidae). Biological Invasions, 17(2), 537–544. 10.1007/s10530-014-0762-6

[eva13694-bib-0065] Pautasso, M. , Aas, G. , Queloz, V. , & Holdenrieder, O. (2013). European ash (*Fraxinus excelsior*) dieback – A conservation biology challenge. Biological Conservation, 158, 37–49. 10.1016/j.biocon.2012.08.026

[eva13694-bib-0066] Pliūra, A. , Lygis, V. , Suchockas, V. , & Bartkevičius, E. (2011). Performance of twenty four European *Fraxinus excelsior* populations in three Lithuanian progeny trials with a special emphasis on resistance to *Chalara fraxinea* . Baltic Forestry, 17, 17–34.

[eva13694-bib-0067] Plumb, W. J. , Coker, T. L. R. , Stocks, J. J. , Woodcock, P. , Quine, C. P. , Nemesio‐Gorriz, M. , & Buggs, R. J. A. (2020). The viability of a breeding programme for ash in the British Isles in the face of ash dieback. Plants, People, Planet, 2(1), 29–40. 10.1002/ppp3.10060

[eva13694-bib-0068] Poland, J. , Endelman, J. , Dawson, J. , Rutkoski, J. , Wu, S. , Manes, Y. , & Jannink, J.‐L. (2012). Genomic selection in wheat breeding using genotyping‐by‐sequencing. The Plant Genome, 5(3), 2012. 10.3835/plantgenome2012.06.0006

[eva13694-bib-0069] Purcell, S. , Neale, B. , Todd‐Brown, K. , Thomas, L. , Ferreira, M. A. R. , Bender, D. , Maller, J. , Sklar, P. , de Bakker, P. I. , Daly, M. J. , & Sham, P. C. (2007). PLINK: A tool set for whole‐genome association and population‐based linkage analyses. The American Journal of Human Genetics, 81(3), 559–575. 10.1086/519795 17701901 PMC1950838

[eva13694-bib-0070] Raj, A. , Stephens, M. , & Pritchard, J. K. (2014). fastSTRUCTURE: Variational inference of population structure in large SNP data sets. Genetics, 197(2), 573–589. 10.1534/genetics.114.164350 24700103 PMC4063916

[eva13694-bib-0071] Resende, M. D. V. , Resende, M. F. R. , Sansaloni, C. P. , Petroli, C. D. , Missiaggia, A. A. , Aguiar, A. M. , Abad, J. M. , Takahashi, E. K. , Rosado, A. M. , Faria, D. A. , Pappas, G. J. , Kilian, A. , & Grattapaglia, D. (2012). Genomic selection for growth and wood quality in eucalyptus: Capturing the missing heritability and accelerating breeding for complex traits in forest trees. New Phytologist, 194(1), 116–128. 10.1111/j.1469-8137.2011.04038.x 22309312

[eva13694-bib-0072] Resende, M. F. , Muñoz, P. , Resende, M. D. , Garrick, D. J. , Fernando, R. L. , Davis, J. M. , Jokela, E. J. , Martin, T. A. , Peter, G. F. , & Kirst, M. (2012). Accuracy of genomic selection methods in a standard data set of loblolly pine (*Pinus taeda* L.). Genetics, 190(4), 1503–1510. 10.1534/genetics.111.137026 22271763 PMC3316659

[eva13694-bib-0073] Roloff, A. (1988). Morphologie der Kronenentwicklung von *Fagus sylvatica* L. (Rotbuche) unter besonderer Berücksichtigung neuartiger Veränderungen: II. Strategie der Luftraumeroberung und Veränderungen durch Umwelteinflüsse. Flora, 180(3), 297–338. 10.1016/S0367-2530(17)30325-0

[eva13694-bib-0074] Roloff, A. (2001). Baumkronen: Verständnis und praktische Bedeutung eines komplexen Naturphänomens. Ulmer.

[eva13694-bib-0075] Sahraei, S. E. , Cleary, M. , Stenlid, J. , Brandström Durling, M. , & Elfstrand, M. (2020). Transcriptional responses in developing lesions of European common ash (*Fraxinus excelsior*) reveal genes responding to infection by *Hymenoscyphus fraxineus* . BMC Plant Biology, 20(1), 455. 10.1186/s12870-020-02656-1 33023496 PMC7541206

[eva13694-bib-0076] Santini, A. , Ghelardini, L. , de Pace, C. , Desprez‐Loustau, M. L. , Capretti, P. , Chandelier, A. , Cech, T. , Chira, D. , Diamandis, S. , Gaitniekis, T. , Hantula, J. , Holdenrieder, O. , Jankovsky, L. , Jung, T. , Jurc, D. , Kirisits, T. , Kunca, A. , Lygis, V. , Malecka, M. , … Stenlid, J. (2013). Biogeographical patterns and determinants of invasion by forest pathogens in Europe. New Phytologist, 197(1), 238–250. 10.1111/j.1469-8137.2012.04364.x 23057437

[eva13694-bib-0077] Skovsgaard, J. P. , Wilhelm, G. J. , Thomsen, I. M. , Metzler, B. , Kirisits, T. , Havrdová, L. , & Clark, J. (2017). Silvicultural strategies for *Fraxinus excelsior* in response to dieback caused by *Hymenoscyphus fraxineus* . Forestry: An International Journal of Forest Research, 90(4), 455–472. 10.1093/forestry/cpx012

[eva13694-bib-0078] Slavov, G. T. , Nipper, R. , Robson, P. , Farrar, K. , Allison, G. G. , Bosch, M. , Clifton‐Brown, J. C. , Donnison, I. S. , & Jensen, E. (2014). Genome‐wide association studies and prediction of 17 traits related to phenology, biomass and cell wall composition in the energy grass *Miscanthus sinensis* . New Phytologist, 201(4), 1227–1239. 10.1111/nph.12621 24308815 PMC4284002

[eva13694-bib-0079] Sniezko, R. A. , Johnson, J. S. , Reeser, P. , Kegley, A. , Hansen, E. M. , Sutton, W. , & Savin, D. P. (2020). Genetic resistance to *Phytophthora lateralis* in port‐Orford‐cedar (*Chamaecyparis lawsoniana*) – Basic building blocks for a resistance program. Plants, People, Planet, 2(1), 69–83. 10.1002/ppp3.10081

[eva13694-bib-0080] Sniezko, R. A. , & Koch, J. (2017). Breeding trees resistant to insects and diseases: Putting theory into application. Biological Invasions, 19(11), 3377–3400. 10.1007/s10530-017-1482-5

[eva13694-bib-0081] Sollars, E. S. A. , Harper, A. L. , Kelly, L. J. , Sambles, C. M. , Ramirez‐Gonzalez, R. H. , Swarbreck, D. , Kaithakottil, G. , Cooper, E. D. , Uauy, C. , Havlickova, L. , Worswick, G. , Studholme, D. J. , Zohren, J. , Salmon, D. L. , Clavijo, B. J. , Li, Y. , He, Z. , Fellgett, A. , McKinney, L. , … Buggs, R. J. (2017). Genome sequence and genetic diversity of European ash trees. Nature, 541(7636), 212–216. 10.1038/nature20786 28024298

[eva13694-bib-0082] Soro, A. , Lenz, P. , Roussel, J.‐R. , Larochelle, F. , Bousquet, J. , & Achim, A. (2022). The phenotypic and genetic effects of drought‐induced stress on apical growth, ring width, wood density and biomass in white spruce seedlings. New Forests, 54, 789–811. 10.1007/s11056-022-09939-5

[eva13694-bib-0083] Spindel, J. E. , Begum, H. , Akdemir, D. , Collard, B. , Redoña, E. , Jannink, J. L. , & McCouch, S. (2016). Genome‐wide prediction models that incorporate de novo GWAS are a powerful new tool for tropical rice improvement. Heredity, 116(4), 395–408. 10.1038/hdy.2015.113 26860200 PMC4806696

[eva13694-bib-0084] Stener, L.‐G. (2013). Clonal differences in susceptibility to the dieback of *Fraxinus excelsior* in southern Sweden. Scandinavian Journal of Forest Research, 28(3), 205–216. 10.1080/02827581.2012.735699

[eva13694-bib-0085] Stocks, J. J. , Metheringham, C. L. , Plumb, W. J. , Lee, S. J. , Kelly, L. J. , Nichols, R. A. , & Buggs, R. J. A. (2019). Genomic basis of European ash tree resistance to ash dieback fungus. Nature Ecology & Evolution, 3(12), 1686–1696. 10.1038/s41559-019-1036-6 31740845 PMC6887550

[eva13694-bib-0086] Tubby, K. V. , & Webber, J. F. (2010). Pests and diseases threatening urban trees under a changing climate. Forestry: An International Journal of Forest Research, 83(4), 451–459. 10.1093/forestry/cpq027

[eva13694-bib-0087] Turner, S. D. (2014). Qqman: An R Package for Visualizing GWAS Results Using Q‐Q and Manhattan Plots. bioRxiv, 20, 5165. 10.1101/005165

[eva13694-bib-0088] Varshney, R. K. , Graner, A. , & Sorrells, M. E. (2005). Genomics‐assisted breeding for crop improvement. Trends in Plant Science, 10(12), 621–630. 10.1016/j.tplants.2005.10.004 16290213

[eva13694-bib-0089] Vázquez‐Lobo, A. , de la Torre, A. R. , Martínez‐García, P. J. , Vangestel, C. , Wegzryn, J. L. , Ćalić, I. , Burton, D. , Davis, D. , Kinloch, B. , Vogler, D. , & Neale, D. B. (2017). Finding loci associated to partial resistance to white pine blister rust in sugar pine (*Pinus lambertiana Dougl*.). Tree Genetics & Genomes, 13(5), 108–117. 10.1007/s11295-017-1190-4

[eva13694-bib-0100] Wang, N. , Thomson, M. , Bodles, W. J. A. , Crawford, R. M. M. , Hunt, H. V. , Featherstone, A. W. , Pellicer, J. , & Buggs, R. J. A. (2012). Genome sequence of dwarf birch (*Betula nana*) and cross‐species RAD markers. Molecular Ecology, 22(11), 3098–3111. 10.1111/mec.12131 23167599

[eva13694-bib-0090] Wang, Q. , Yu, Y. , Zhang, Q. , Zhang, X. , Huang, H. , Xiang, J. , & Li, F. (2019). Evaluation on the genomic selection in *Litopenaeus vannamei* for the resistance against Vibrio parahaemolyticus. Aquaculture, 505, 212–216. 10.1016/j.aquaculture.2019.02.055

[eva13694-bib-0091] Westbrook, J. W. , Zhang, Q. , Mandal, M. K. , Jenkins, E. V. , Barth, L. E. , Jenkins, J. W. , Grimwood, J. , Schmutz, J. , & Holliday, J. A. (2020). Optimizing genomic selection for blight resistance in American chestnut backcross populations: A trade‐off with American chestnut ancestry implies resistance is polygenic. Evolutionary Applications, 13(1), 31–47. 10.1111/eva.12886 31892942 PMC6935594

[eva13694-bib-0092] Wickham, H. (2016). ggplot2: Elegant graphics for data analysis. Springer Cham.

[eva13694-bib-0093] Wientjes, Y. C. J. , Veerkamp, R. F. , & Calus, M. P. L. (2013). The effect of linkage disequilibrium and family relationships on the reliability of genomic prediction. Genetics, 193(2), 621–631. 10.1534/genetics.112.146290 23267052 PMC3567749

[eva13694-bib-0094] Yamashita, H. , Uchida, T. , Tanaka, Y. , Katai, H. , Nagano, A. J. , Morita, A. , & Ikka, T. (2020). Genomic predictions and genome‐wide association studies based on RAD‐seq of quality‐related metabolites for the genomics‐assisted breeding of tea plants. Scientific Reports, 10(1), 17480. 10.1038/s41598-020-74623-7 33060786 PMC7562905

[eva13694-bib-0095] Yu, J. , Pressoir, G. , Briggs, W. H. , Vroh Bi, I. , Yamasaki, M. , Doebley, J. F. , McMullen, M. , Gaut, B. S. , Nielsen, D. M. , Holland, J. B. , Kresovich, S. , & Buckler, E. S. (2006). A unified mixed‐model method for association mapping that accounts for multiple levels of relatedness. Nature Genetics, 38(2), 203–208. 10.1038/ng1702 16380716

[eva13694-bib-0096] Zhang, H.‐L. , Jiang, W.‐L. , Liu, R. , Zhou, Y. , & Zhang, Y. (2019). Organic degradation and extracellular products of pure oxygen aerated activated sludge under different F/M conditions. Bioresource Technology, 279, 189–194. 10.1016/j.biortech.2019.01.130 30735927

[eva13694-bib-0097] Zhang, Z. , Ober, U. , Erbe, M. , Zhang, H. , Gao, N. , He, J. , Li, J. , & Simianer, H. (2014). Improving the accuracy of whole genome prediction for complex traits using the results of genome wide association studies. PLoS One, 9(3), e93017. 10.1371/journal.pone.0093017 24663104 PMC3963961

[eva13694-bib-0098] Zhao, Y. , Gowda, M. , Liu, W. , Würschum, T. , Maurer, H. P. , Longin, F. H. , Ranc, N. , & Reif, J. C. (2012). Accuracy of genomic selection in European maize elite breeding populations. Theoretical and Applied Genetics, 124(4), 769–776. 10.1007/s00122-011-1745-y 22075809

[eva13694-bib-0099] Zheng, X. , Levine, D. , Shen, J. , Gogarten, S. M. , Laurie, C. , & Weir, B. S. (2012). A high‐performance computing toolset for relatedness and principal component analysis of SNP data. Bioinformatics, 28(24), 3326–3328. 10.1093/bioinformatics/bts606 23060615 PMC3519454

