# Supplementary material for: Genomic prediction of resistance to Hymenoscyphus fraxineus in common ash (Fraxinus excelsior L.) populations
Source: Evol Appl. 2024 May 3;17(5):e13694. doi: 10.1111/eva.13694 (PMC11069026; doi:10.1111/eva.13694)
Supplement: Supplementary file 2 — Figures S1–S2. [file EVA-17-e13694-s001.docx]

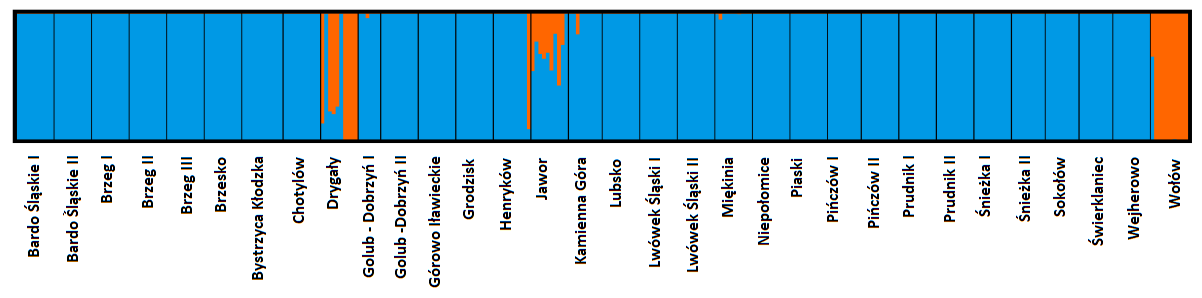

**Figure S1**. Graphical representation of the membership coefficients per individual at K = 2 clusters inferred from fastSTRUCTURE.

| 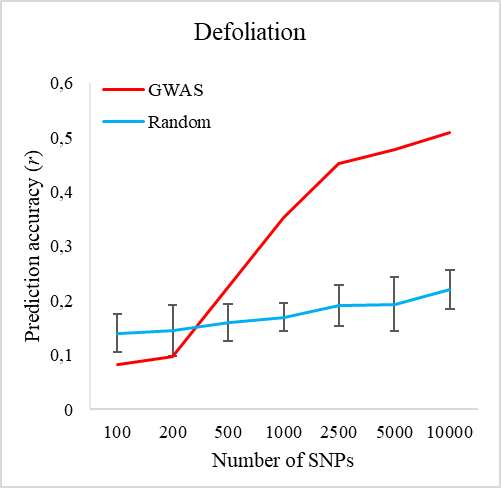 | 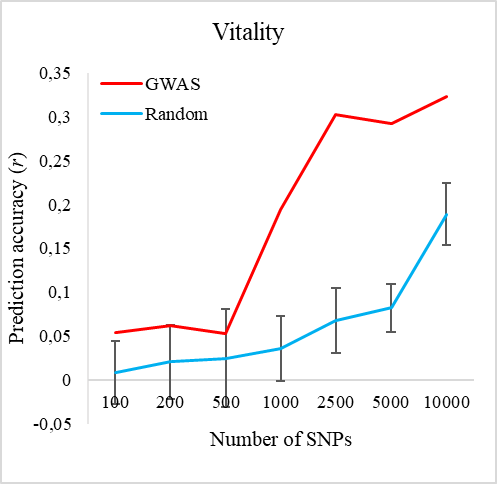 |
| --- | --- |
|  | 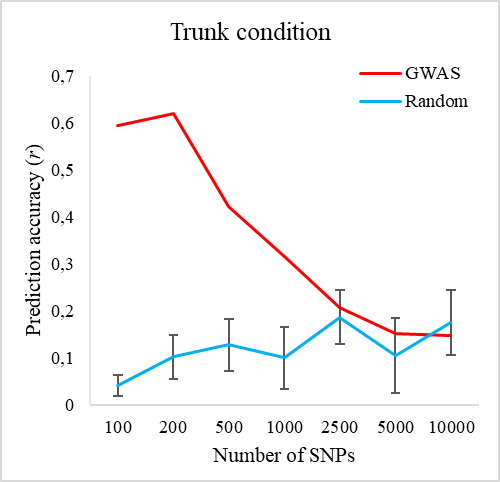 |
| 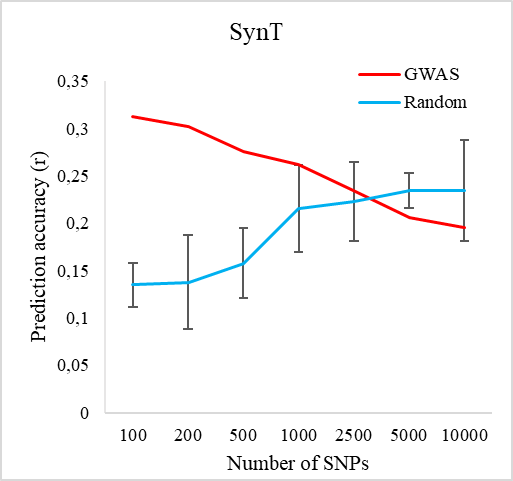 |  |

**Figure S2.** Genomic prediction accuracy, calculated as the correlation of GEBV in dataset B with health status indicators (defoliation, vitality, Syn, trunk condition, and SynT). Error bars for random datasets represent the means ± standard error (based on the selection of random SNP datasets performed 10 times).
